# Text-Book for Nurses

**Published:** 1912-12

**Authors:** 


**Text-Book for Nurses.** By E. W. Hey Groves, M.S., F.R.C.S., and J. M. Fortescue-Brickdale, M.A., M.D. Pp. xxiv, 407. London: Oxford Medical Publications. 1912. Price, 12s. 6d. net.

It is always very difficult to know just how much information it is wise to give to nurses, nevertheless a helper who knows her work, and can take an intelligent interest in it is far more valuable than one who merely follows some rule of thumb, and the text-book under review is well calculated to train the best type of nurse. Although they have had to create their own precedents for the most part, the authors have displayed admirable judgment in deciding what to include and how much to say about it. They are singularly successful in putting their teaching into such language as may be understood by those for whom they write, and this is a genuine triumph. Needless to say, their accounts of the various diseases are thoroughly reliable and up to date.

Some of the anatomical descriptions present an unfamiliar appearance, because the authors have seen fit to adopt the Basle nomenclature, and it will be a matter of opinion whether the time is ripe for this. The physiological section, though well written, is scarcely as up to date as the remainder of the book. The respiratory centre is said to be stirred into activity by vagus influence instead of by carbon dioxide in the blood (p. 91), the origin of leucocytes from bone-marrow is not mentioned (p. 83), and fats are said to be absorbed partly as an emulsion, whereas the modern teaching attributes supreme importance to the action of fat-splitting ferments. Proteins are said to pass through the intestinal wall as peptones, not as amino-acids. Purists in nomenclature as they are, the writers still call proteins by the old name of proteids. Several misprints (*e.g. renin, mucous*, p. 102) occur in this section.

The section on the preparation of a patient for an anæsthetic might be extended a little with advantage by mentioning the value of long warm stockings, and the usefulness of a split nightdress. It would be valuable also to describe the arrangement of a room in a private house. Although we do not personally object to the authors' permission to give a cup of tea two hours before an anæsthetic, we feel sure that nurses would get into trouble with many surgeons if they acted on this advice.

We suggest also that in future editions, which will undoubtedly be called for, the symptoms of overdosing with ordinary drugs should be given.

The illustrations are very numerous, admirably chosen, and (with a few exceptions, figs. 139, 184, 189) beautifully executed. They will add immensely to the teaching value of the book.

